# Combining Network Pharmacology with Molecular Docking for Mechanistic Research on Thyroid Dysfunction Caused by Polybrominated Diphenyl Ethers and Their Metabolites

**DOI:** 10.1155/2021/2961747

**Published:** 2021-11-17

**Authors:** Qiaoyu He, Xiaopeng Chen, Jing Liu, Chunxia Li, Hong Xing, Yumeng Shi, Qian Tang

**Affiliations:** State Key Laboratory of Component-Based Chinese Medicine, Tianjin University of Traditional Chinese Medicine, Tianjin 301617, China

## Abstract

Network pharmacology was used to illuminate the targets and pathways of polybrominated diphenyl ethers (PBDEs) causing thyroid dysfunction. A protein-protein interaction (PPI) network was constructed. Molecular docking was applied to analyze PBDEs and key targets according to the network pharmacology results. A total of 247 targets were found to be related to 16 PBDEs. Ten key targets with direct action were identified, including the top five PIK3R1, MAPK1, SRC, RXRA, and TP53. Gene Ontology (GO) functional enrichment analysis identified 75 biological items. Kyoto Encyclopedia of Genes and Genomes (KEGG) pathway analysis identified 62 pathways mainly related to the regulation of the thyroid hormone signaling pathway, MAPK signaling pathway, PI3K-Akt signaling, pathways in cancer, proteoglycans in cancer, progesterone-mediated oocyte maturation, and others. The molecular docking results showed that BDE-99, BDE-153, 5-OH-BDE47, 5′-OH-BDE99, 5-BDE47 sulfate, and 5′-BDE99 sulfate have a good binding effect with the kernel targets. PBDEs could interfere with the thyroid hormone endocrine through multiple targets and biological pathways, and metabolites demonstrated stronger effects than the prototypes. This research provides a basis for further research on the toxicological effects and molecular mechanisms of PBDEs and their metabolites. Furthermore, the application of network pharmacology to the study of the toxicity mechanisms of environmental pollutants provides a new methodology for environmental toxicology.

## 1. Introduction

Polybrominated diphenyl ethers (PBDEs) are persistent organic pollutants that exist widely in the environment. They are widely used in daily life and have been added as fire extinguishing materials to various polymer resins and plastics, furniture, television sets, interior decoration, and others [1]. PBDEs are ubiquitous in the atmosphere, soil, animals, and plants, including blood, fat, food, and even milk [2, 3]. The distribution and accumulation of PBDEs pose a threat to human health.

The toxicological mechanism of PBDEs is the focus of research. The chemical general formula is C_12_H*_n_*Br_(10-*n*)_O. The structure of PBDEs is similar to that of thyroid hormones, resulting in binding to thyroid receptors (TRs) and interference with the thyroid endocrine [4]. Meanwhile, the hydroxylated metabolites of PBDEs have a stronger binding affinity to thyroid hormone transporters (TBPA) in serum, thus making more thyroxine (T4) available in a free mode. The binding affinity of OH-PBDEs to human transthyretin (TTR) and thyroid-binding globulin (TBG) is higher than that of its prototype [5]. Similarly, PBDE sulfate metabolites interfere with the TH system by binding to TH transporters or TRs [6]. Most toxicity mechanism studies mainly focus on a single monomer. However, multiple PBDE homologues exist simultaneously in the environment and organisms, showing combined action. Therefore, it is necessary to study the mechanism of toxicity of multiple homologues simultaneously.

The pathogenesis of thyroid dysfunction is complicated, and influencing factors include sex, smoking, lifestyles, and variation in physiological status, such as that of the immune system, as well as other pathological indicators, such as glucose tolerance and cholesterol levels [7]. Single targets and pathways have difficulty reflecting the pathological state of thyroid dysfunction, which brings great challenges to the study of the mechanism of PBDE interference on thyroid hormone secretion.

Network pharmacology is a strong tool for studying the synergistic effect of multiple components and targets [8, 9]. In 2007, the British pharmacologist Hopkins [10] proposed the concept of network pharmacology to regulate the whole biological network, which could clarify the relationship between drug targets and disease-related proteins [11]. Based on the theory of systems biology, a virtual computer method is used to visually analyze the whole biological system to effectively predict relevant targets and pathways. Applying network pharmacology to environmental toxicology and looking for the key targets and pathways of PBDEs acting on thyroid hormone endocrine disruption in complex systems can reveal the mechanism of action based on multiple components and targets. It also provides a basis for further study of the pathological mechanism of thyroid endocrine disruption caused by small molecular organic pollutants.

Molecular docking is a major computing technology in the field of computer-aided drug research. In the early 1980s, Kuntz et al. proposed the application of molecular docking in drug discovery and biology [12]. As one of the widely used virtual screening methods, the main purpose is to predict the binding mode and affinity between ligands and receptors with pertinence and specificity.

This study used network pharmacology in drug research and development for reference and applied it to the study of environmental toxicology to explore the mechanism of 16 PBDEs on thyroid endocrine disruption. Furthermore, molecular docking was used to verify the binding of active homologues and key targets. The methodology is aimed at studying the toxicological mechanism of PBDEs on thyroid dysfunction at the target and pathway levels from the integral action of multiple homologues.

**Figure 1:**
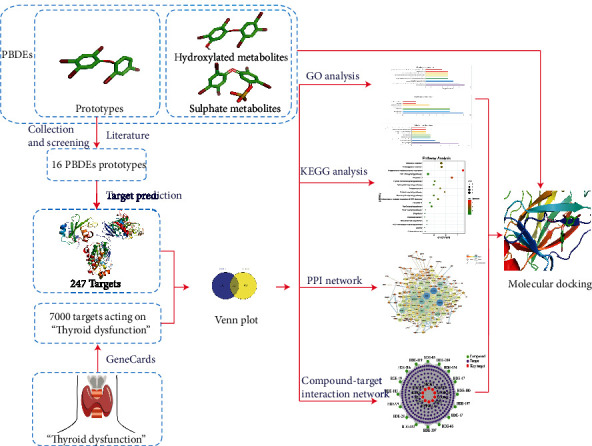
General workflow of network pharmacology and molecular docking in the present study.

In our study, the potential effects and mechanisms of PBDE prototypes influencing thyroid dysfunction were analyzed by network pharmacology. Molecular interactions between PBDE prototypes, metabolites, and target receptors related to thyroid dysfunction were studied by molecular docking, which is shown in Figure 1.

## 2. Materials

### 2.1. Network Pharmacology Database and Software

Four databases were used to search target genes related to PBDEs, including the STITCH database (http://stitch.embl.de/), Swiss Target Prediction database (http://www.swisstargetprediction.ch/), SEA database (http://sea.edbc.org/), and Pharmmapper database (http://www.lilab-ecust.cn/pharmmapper). Then, the GeneCards database (http://www.genecards.org/) was used to search thyroid dysfunction-related genes. The Venny 2.1.0 online website (http://bioinfogp.cnb.csic.es/tools/venny/index.html) was used to obtain a Venn diagram. The STRING database (http://string-db.org/) was used to predict potential PPI networks. Cytoscape software (version 3.6.1) was used to draw the PBDE-thyroid dysfunction interaction network and screen key targets. Finally, the DAVID 6.8 database (http://david.ncifcrf.gov/) was used to perform Gene Ontology (GO) functional enrichment analysis and Kyoto Encyclopedia of Genes and Genomes (KEGG) pathway analysis.

### 2.2. Molecular Docking Database and Software

The RCSB Protein Data Bank (http://www.rcsb.org/) was used to download the crystal structures of protein targets. Chem3D 19.0 software was used to create PBDE chemical structures and minimize their energy. AutoDock Tools 1.5.6 [13] and AutoDock Vina [14] were used for the pretreatment and docking of proteins and ligands. Finally, PyMOL [15] software and LigPlot [16] software were used to visualize the results.

## 3. Experimental Method

### 3.1. Network Pharmacology

#### 3.1.1. PBDE Information

Search PBDEs ubiquitous in the environment and human body and related to thyroid disorders in the literature through Web of Science, and the retrieval time remains the default [17–21]. BDE-17, BDE-28, BDE-47, BDE-49, BDE-66, BDE-85, BDE-99, BDE-100, BDE-119, BDE-153, BDE-154, BDE-183, BDE-197, BDE-206, BDE-207, and BDE-209 were selected. The PubChem database (https://pubchem.ncbi.nlm.nih.gov/) and ChemSpider database (http://www.chemspider.com/) were used to acquire the 3D structures and the corresponding SMILES structural formulas of the compounds.

#### 3.1.2. Screening PBDE-Related Targets

The SMILES structural formulas of the compounds in 3.1.1 were input into the STITCH (http://stitch.embl.de/), Swiss Target Prediction (http://www.swisstargetprediction.ch/), and SEA (http://sea.edbc.org/) databases. “Homo sapiens” was selected as the species to search for the desired targets. Similarly, the 3D structures of PBDEs were uploaded to the Pharmmapper database (http://www.lilab-ecust.cn/pharmmapper/), and “Homo sapiens” was selected as the Select Targets Set option.

#### 3.1.3. Screening Thyroid Dysfunction-Related Targets

The GeneCards database (https://www.genecards.org/) was used to screen targets related to thyroid dysfunction with “Thyroid dysfunction” as the keyword.

#### 3.1.4. Screening PBDE-Thyroid Dysfunction Common Targets

The gene names corresponding to PBDE- and thyroid dysfunction-related targets were intersected using the Venny 2.1.0 online website to obtain the common targets.

#### 3.1.5. Construction of a PPI Network

The common targets of PBDE-thyroid dysfunction were imported into the STRING database (https://string-db.org/), and the species was selected as “Homo sapiens.” The protein interaction network was constructed based on the nodes with a score greater than 0.9, while the discrete nodes were hidden. Then, the network was imported into Cytoscape 3.6.1 to construct a PPI network.

#### 3.1.6. Screening Key Target Proteins in the PPI Network

The protein interaction network diagram was imported into Cytoscape 3.6.1, and the network analyzer function was used to visually analyze the network. Based on the preliminary screening of 2 times the median of “Degree,” a new network was created with the selected kernel nodes. Then, the medians of “Degree,” “Closeness Centrality,” and “Betweenness Centrality” were used for further selection. The nodes meeting the above conditions were selected as the key targets of the PBDE-thyroid dysfunction interaction network.

#### 3.1.7. GO Enrichment and KEGG Pathway Analyses

The key targets obtained in Section 3.1.6 were imported into the DAVID 6.8 database (https://david.ncifcrf.gov/), and GO enrichment and KEGG pathway analyses were carried out with *P* < 0.05 as the screening condition.

### 3.2. Molecular Docking

Based on the PBDE detected in the environment, BDE-47, BDE-99, BDE-100, BDE-153, and BDE-154 were selected, which are found at relatively high levels in the human body, as reported in the literature [22, 23]. The hydroxyl and sulfate metabolites BDE-47 and BDE-99 were also selected for molecular docking verification, including 6-OH-BDE47, 5-OH-BDE47, 3-OH-BDE47, 5-OH-BDE99, 5′-OH-BDE99, 6-BDE47 sulfate, 5-BDE47 sulfate, 3-BDE47 sulfate, 5-BDE99 sulfate, and 5′-BDE99 sulfate [24–26]. The 3D structure of PBDEs was drawn by Chem3D 19.0 software and optimized by the MM2 function to obtain the optimum structure. The protein crystal structures of the kernel targets were downloaded from the PDB database (http://www.rcsb.org/), and waters and ligands were removed by PyMOL software. The protein crystal structure was imported into AutoDock Tools 1.5.6 to hydrogenate, calculate the total charge, and add atomic types. Finally, the processed protein and PBDEs were docked with AutoDock Vina to verify the molecular binding. The visualization of docking results was realized by using PyMOL and LigPlot software.

## 4. Results

### 4.1. Network Pharmacology

#### 4.1.1. PBDE Information

The ChemSpider database (http://www.chemspider.com/) and PubChem database (http://pubchem.ncbi.nlm.nih.gov/) were used to determine the SMILES structural formulas of the compounds (Supplementary Table S1). The PBDE general structure is shown (Supplementary Figure S1).

#### 4.1.2. Screening PBDE-Related Targets

A total of 208 related targets were collected based on 3D structures of 16 PBDEs in the Pharmmapper database. Based on the SMILES structural formulas, 25 and 9 related targets were obtained from the Swiss Target Prediction and SEA databases, respectively. Similarly, the SMILES structural formula was imported into the STITCH database, and 19 related targets were identified. In total, 247 related target genes were obtained by combining the above targets and deleting duplicates.

#### 4.1.3. Screening Thyroid Dysfunction-Related Targets

Through the GeneCards database, 7000 disease targets were screened with “Thyroid dysfunction” as the keyword.

#### 4.1.4. Screening PBDE-Thyroid Dysfunction Common Targets

The PBDE targets were intersected with disease targets using the Venny 2.1.0 online website, and 207 intersection targets were obtained (Supplementary Figure S2).

#### 4.1.5. Construction of the PPI Network

The 207 intersection targets obtained by Venny 2.1.0 were imported into the STRING database. The preliminary screening was carried out at twice the median of “Degree.” The medians of “Degree,” “Closeness Centrality,” and “Betweenness Centrality” were used for further filtering, and the graph contained 162 interacting nodes and 610 interacting edges (Supplementary Figure S3).

#### 4.1.6. Screening Key Target Proteins in the PPI Network

**Figure 2:**
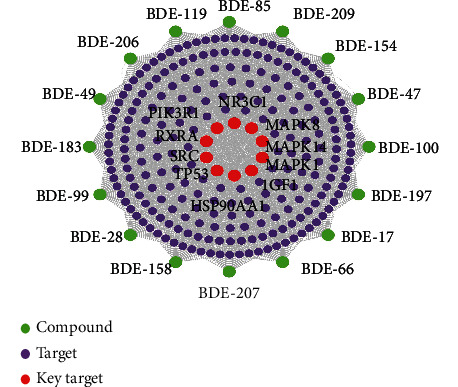
PBDE-thyroid dysfunction interaction network and kernel targets.

By using the network analyzer function, 31 related nodes and 161 target-to-target relationships were obtained. The key target protein was obtained by a second round of screening with the medians of “Degree,” “Closeness Centrality,” and “Betweenness Centrality.” The rank by degree value was PIK3R1, MAPK1, SRC, RXRA, TP53, MAPK14, HSP90AA1, MAPK8, NR3C1, and IGF1. The PBDE-thyroid dysfunction interaction network and kernel targets are shown in Figure 2. Details of the compound targets are shown (Supplementary Table S2).

#### 4.1.7. GO Enrichment Analysis

**Figure 3:**
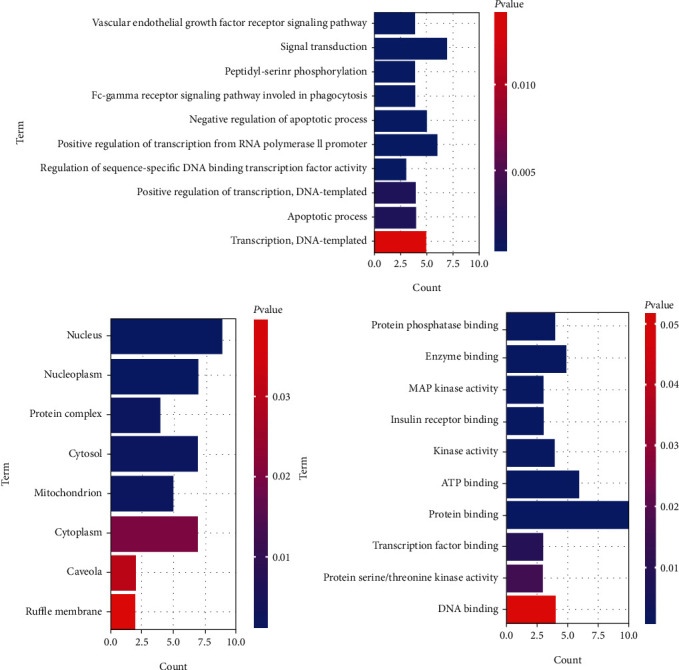
BP: biological process; CC: cellular component; MF: molecular function. *P* < 0.05.

The 10 key target proteins were introduced into the DAVID 6.8 database. Seventy-four GO items were obtained by GO enrichment analysis (*P* < 0.05). Among them, 53 items were associated with the biological process (BP), 8 items were associated with the cellular component (CC), and 13 items were associated with the molecular function (MF), accounting for 71.6%, 10.8%, and 17.6% of the total GO enrichment, respectively. The top 10 items in BP, CC, and MF are shown in Figure 3. The GO enrichment analysis results showed that the top 10 targets were mainly involved in biological processes such as signal transduction, positive regulation of transcription from the RNA polymerase II promoter, negative regulation of the apoptotic process, and transcription, DNA-templated.

#### 4.1.8. KEGG Pathway Analysis

**Figure 4:**
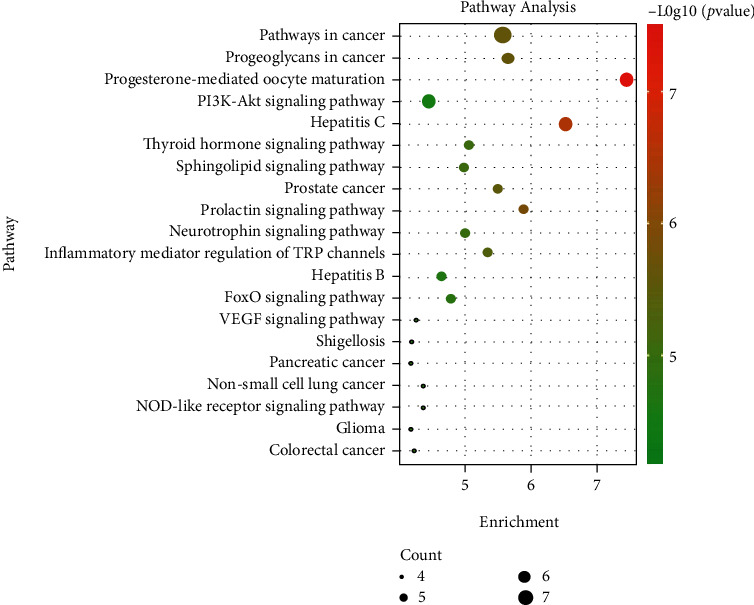
Bubble chart of the KEGG pathway analysis. The size of the dot represents the number of genes involved in the process. The larger the point, the greater the number of genes. The color depth represents the *P* value, and the darker the color, the smaller the *P* value.

The key targets were imported into the DAVID 6.8 database. The KEGG pathway analysis was carried out under the screening condition of *P* < 0.05, and 62 entries were obtained. The top 20 items are listed in Figure 4. In the top 20 items, the thyroid hormone signaling pathway included target proteins PIK3R1, MAPK1, SRC, RXRA, and TP53, which were the top 5 ranking targets in the kernel target analysis in Section 4.1.6. It is suggested that PBDEs interfere with the thyroid endocrine in organisms by acting on the five targets in the thyroid hormone signal pathway. A detailed map of the thyroid hormone signaling pathway is shown (Supplementary Figure S4).

### 4.2. Molecular Docking

**Figure 5:**
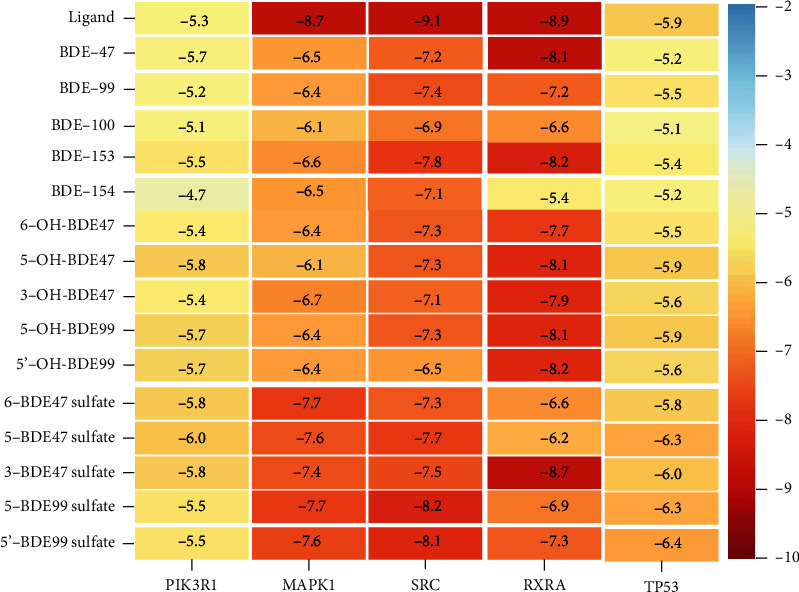
The docking combination between the PBDEs and the targets.

To further verify the reliability of network pharmacology prediction, molecular docking was used to explore the binding of PBDEs and their metabolites to thyroid hormone endocrine disruption targets. BDE-47, BDE-99, BDE-100, BDE-153, and BDE-154 were selected because of the high frequency and content detected in human tissues. The hydroxylated metabolites of BDE-47 and BDE-99 (6-OH-BDE47, 5-OH-BDE47, 3-OH-BDE47, 5-OH-BDE99, and 5′-OH-BDE99) and the sulfate metabolites (6-BDE47 sulfate, 5-BDE47 sulfate, 3-BDE47 sulfate, 5-BDE99 sulfate, and 5′-BDE99 sulfate) were also selected. The top 5 kernel targets screened by network pharmacology, including PIK3R1 [27] (PDB code: 1H9O), MAPK1 [28] (PDB code: 1TVO), SRC [29] (PDB code: 6E6E), RXRA [30] (PDB code: 1MVC), and TP53 [31] (PDB code: 6GGA), were used as docking targets with PBDEs and metabolites to verify the accuracy of the prediction. Information on targets and ligands involved in molecular docking is shown (Supplementary Table S3). The docking combination between the PBDEs and the targets is shown in Figure 5. The binding energy between the PBDEs and the targets is an important index to reflect the docking capacity. It is generally recognized that binding energy less than -5.0 kcal·mol^−1^ indicates better binding activity between the components and the targets. The smaller the binding energy is, the stronger the binding ability between the component and the target [32, 33]. The molecule with the lowest binding energy in the docking conformation was selected to observe the binding effect by analyzing the intermolecular interaction.

**Figure 6:**
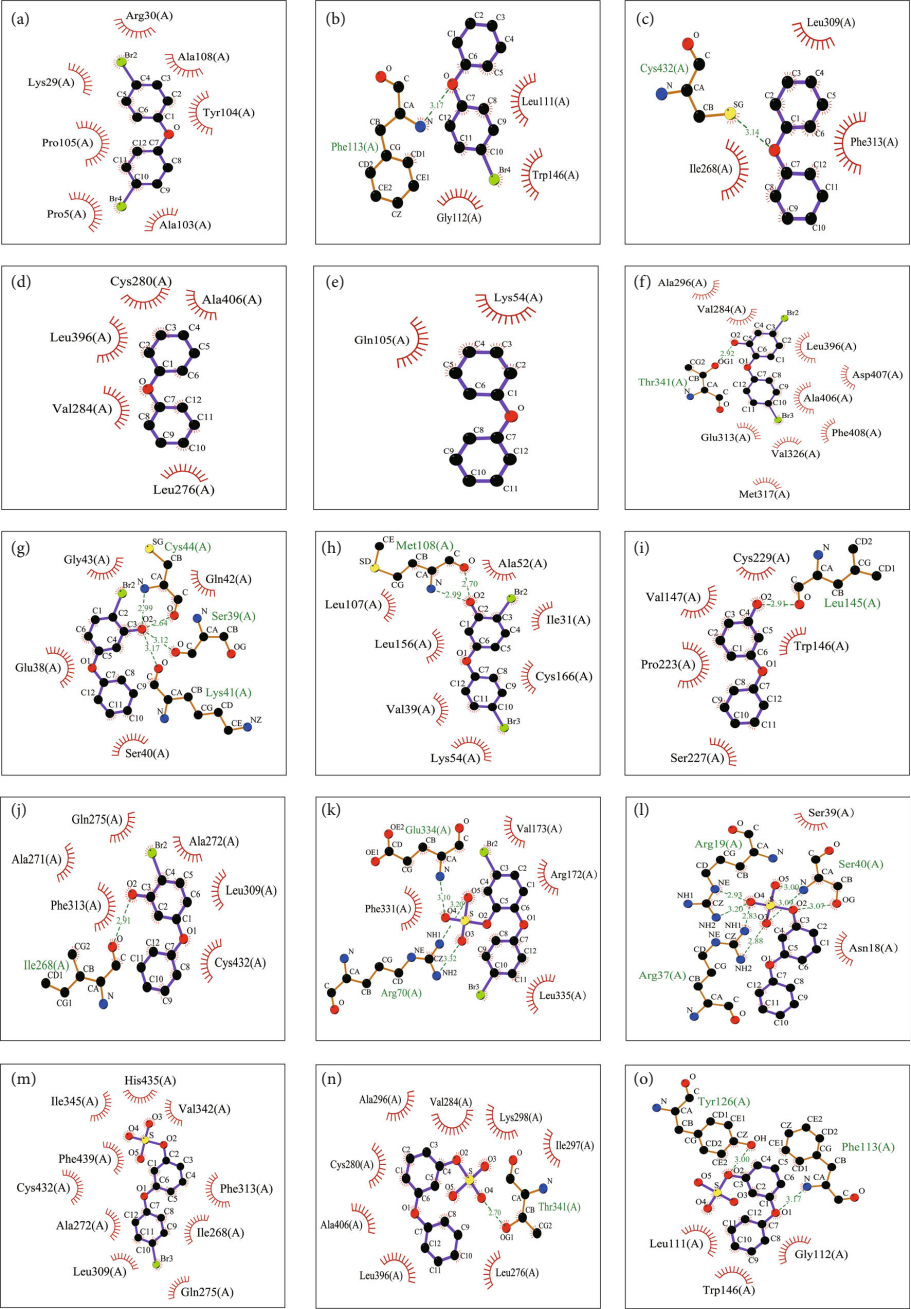
2D visual analysis results of molecular docking of PBDE prototypes, hydroxylated metabolites, and sulfate metabolites with key targets (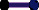 = ligand bond; 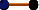 = nonligand bond; 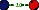 = hydrogen bond and its length; 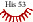 = nonligand residues involved in hydrophobic contact(s); 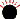 = corresponding atoms involved in hydrophobic contact(s)).

The 2D visual analysis results of the molecular docking of PBDE prototypes, hydroxylated metabolites, and sulfate metabolites with key targets are shown in Figure 6. The 3D visual analysis results of molecular docking of PBDE prototypes, hydroxylated metabolites, and sulfate metabolites with key targets are shown (Supplementary Figure S5). The 2D and 3D visual analysis results of molecular docking of natural ligands with key targets are shown (Supplementary Figure S6).

#### 4.2.1. Molecular Docking of PBDE Prototypes with Key Targets

The docking results of PBDE prototypes and key targets showed that the binding energy of BDE-153 docking with RXRA targets was the lowest, which is shown in Figure 5. The docking binding energies of all key targets with PBDE prototypes were less than -5 kcal·mol^−1^. Among them, the docking binding energy of BDE-47 with PIK3R1 was lower than that of ligand PTR. The docking interactions between PBDE prototypes and key targets mainly include hydrogen bond interactions, hydrophobic interactions, and *π*-*π* interactions. Specific descriptions were shown (Supplementary File S1). The docking results of PBDEs are shown in Figures 6(a)–6(e), and BDE-153 is illustrated as an example.

The docking results of BDE-153 and RXRA are shown in Figure 6(c). BDE-153 formed a hydrogen bond interaction with the S on the main chain of amino acid residue Cys432. It also had hydrophobic interactions with the hydrophobic cavity of three amino acid residues, Leu309, Phe313, and Ile268, near the active site. The natural ligand BM6 had hydrophobic interactions with the hydrophobic cavity of eight amino acid residues, Glu453, Thr449, Phe450, Leu301, Val298, Leu294, Val280, and Phe277. The docking binding energy between BDE-153 and RXRA was -8.2 kcal·mol^−1^, which was higher than the docking binding energy of the natural ligand BM6 and RXRA of -8.9 kcal·mol^−1^ and the common standard of -5.0 kcal·mol^−1^.

#### 4.2.2. Molecular Docking of PBDE Hydroxylated Metabolites with Key Targets

The docking results of PBDE hydroxylated metabolites with key targets showed that the docking binding energy of 5′-OH-BDE99 with RXRA targets was the lowest, which is shown in Figure 5. The docking binding energies of all PBDE hydroxyl metabolites with key targets were less than -5 kcal·mol^−1^. Among them, the docking binding energy of 5-OH-BDE47 with PIK3R1 was lower than that of ligand PTR. The docking interactions between PBDE hydroxyl metabolites and key targets mainly include hydrogen bond interactions and hydrophobic interactions. Specific descriptions were shown (Supplementary File S2). The docking results of hydroxylated metabolites are shown in Figures 6(f)–6(j), and 5-OH-BDE47 is illustrated as an example.

The docking results of 5-OH-BDE47 and PIK3R1 are shown in Figure 6(g). The interaction of 5-OH-BDE47 and PIK3R1 included a hydrogen bond interaction with NH and C=O on the main chain of amino acid residue Cys44, a hydrogen bond interaction with C=O on the main chain of Ser39 and Lys41, and hydrophobic interactions with the hydrophobic cavity of four amino acid residues, Gly43, Gln42, Glu38, and Ser40, near the active site. The natural ligand PTR formed three hydrogen bond interactions with the active site of PIK3R1, a hydrogen bond interaction with the NH on the main chain of amino acid residues His85, His88, and Ser77, and hydrophobic interactions with the hydrophobic cavity formed by the four amino acid residues Leu75, Tyr76, Glu81, and Leu84 near the active site. The docking binding energy of 5-OH-BDE47 and PIK3R1 was -5.8 kcal·mol^−1^ less than -5.0 kcal·mol^−1^, and it was lower than the docking binding energy of the natural ligand PTR and PIK3R1 of -5.3 kcal·mol^−1^.

#### 4.2.3. Molecular Docking of PBDE Sulfate Metabolites with Key Targets

The docking results of PBDE sulfate metabolites with key targets showed that the docking binding energy of 3-BDE47 sulfate with RXRA targets was the lowest, which is shown in Figure 5. The docking binding energies of all key targets with PBDE sulfate metabolites were less than -5 kcal·mol^−1^. Among them, the docking binding energy of 5-BDE47 sulfate with PIK3R1 was lower than that of ligand PTR with the target, and the binding energy of 5-BDE99 sulfate with TP53 was lower than that of ligand EY2 with the target. The docking interactions between PBDE sulfate metabolites and key targets mainly include hydrogen bonding and hydrophobic interactions. Specific descriptions are available (Supplementary File S3). The docking results of sulfate metabolites are shown in Figures 6(k)–6(o), and 5-BDE47 sulfate is illustrated as an example.

The docking results of 5-BDE47 sulfate and PIK3R1 are shown in Figure 6(l). 5-BDE47 sulfate formed two hydrogen bonding interactions with the NH and NH_2_ around C6 on the main chain of amino acid residue Arg19 and formed two hydrogen bonding interactions with the two NH_2_ around C6 on the main chain of amino acid residue Arg37. Meanwhile, it formed two hydrogen bonding interactions with the NH and one hydrogen bonding interaction with the OH on the main chain of amino acid residue Ser40. It also had a hydrophobic interaction with the amino acid residues Ser39 and Asn18. The natural ligand PTR formed three hydrogen bond interactions with the active site of PIK3R1, a hydrogen bond interaction with the NH on the main chain of amino acid residues His85, His88, and Ser77, and hydrophobic interactions with the hydrophobic cavity of the four amino acid residues Leu75, Tyr76, Glu81, and Leu84 near the active site. The docking binding energy of 5-BDE47 sulfate and PIK3R1 was -6.0 kcal·mol^−1^ less than -5.0 kcal·mol^−1^ and lower than that of the natural ligand PTR (-5.3 kcal·mol^−1^). The docking interactions between the natural ligand and the key targets mainly include hydrogen bonding and hydrophobic interactions. Specific descriptions are available (Supplementary File S4).

## 5. Discussion

### 5.1. The Effect of PBDEs and Their Metabolites on Thyroid Dysfunction

In thyroid interference, PBDE monomers and mixtures show different toxicities. The method of network pharmacology to study the targets and pathways of PBDEs using a holistic view is consistent with the state of PBDEs in nature and organisms. Therefore, the results can better reflect the overall role of environmental pollutants. Commercial PBDE mixture DE-71 is composed of more than 20 homologues, and BDE-47 and BDE-99 account for approximately 87% of the mixture. The triiodothyronine (T3) level is lower in the body of salmon exposed to the mixture of BDE-47 and BDE-99 than in those exposed to any of the homologues alone at the same concentration of PBDEs [34].

The targets obtained by network pharmacology are applicable for PBDE prototypes and structural analogues such as metabolites. This structural extrapolation is also a characteristic of network pharmacology. Therefore, based on obtaining key targets from PBDE prototypes, the thyroid hormone endocrine interference mechanism of PBDE metabolites was studied further. Compared with the precursor, PBDE metabolites exhibit stronger thyroid hormone interference effects. As the phase I metabolite of PBDEs, OH-PBDEs are more similar in structure to thyroid hormones than the prototype, resulting in a stronger binding ability to TTR. The mechanism by which OH-PBDEs interfere with thyroid hormones can act as an effective competitor for binding to thyroid transporter TTR or directly to TRs, thus interfering with the balance of thyroid hormones in vivo [25, 35]. Studies have confirmed that compared with the corresponding precursors of OH-PBDEs, PBDE sulfate metabolites have a stronger binding capacity for TH transporters and TRs [6].

The number (monomer or mixture) and the metabolic form (hydroxylated or sulfate metabolites) will affect the thyroid hormone interference at different levels, resulting in varying degrees of damage to the body. Therefore, based on monomer research, more attention can be paid to mixtures and hydroxylated and sulfate metabolites to further clarify the toxicity mechanism of the mixture and various metabolites.

### 5.2. Key Targets of Thyroid Function Affected by PBDEs

Multiple key target proteins of PBDEs affecting thyroid function were screened by network pharmacology, indicating that PBDEs affect thyroid endocrine function through multiple targets acting on multiple pathways throughout the whole biological network.

Ten key target proteins were screened, and the top five were PIK3R1, MAPK1, SRC, RXRA, and TP53. PIK3R1 is a phosphatidylinositol-3-kinase regulatory subunit (P85*α*) coding gene related to thyroid function. PIK3R1 RNA could be moderately expressed in the thyroid by regulating the PI3K-Akt signal pathway, which plays an important role in the development of thyroid cancer [36, 37]. MAPK1 is a mitogen-activated protein kinase that is overexpressed in different types of cancer. MAPK1 is a direct target of miR-675 in thyroid papillary carcinoma (PTC) cells and plays an important role in the occurrence and development of PTC by regulating pathological processes [38]. SRC is a member of the family of nonreceptor protein tyrosine kinases, a proto-oncogene that regulates a variety of cellular processes, including growth, migration, and invasion. SRC is the central mediator of the growth and metastasis of thyroid cancer. Dasatinib inhibits the growth and metastasis of thyroid cancer cells by inhibiting the SRC gene [39]. RXRA is retinol-like X receptor alpha, a human nuclear receptor encoded by the RXRA gene. RXRA plays an important role in the occurrence and development of malignant tumors, and its abnormal expression rate in thyroid carcinoma is 66% [40]. TP53 is an important tumor suppressor gene. Mutation of the TP53 gene directly leads to the inactivation of TP53 in approximately half of the tumors. It has been reported that there is a strong correlation between TP53Arg72Pro polymorphism and thyroid cancer [41]. The top five key targets all have a certain relationship with thyroid diseases, which further verifies the reliability of the network pharmacology results.

### 5.3. Pathways Affected by PBDEs

The KEGG pathway analysis shows that pathways affected by PBDEs mainly involve the pathways in cancer, proteoglycans in cancer, progesterone-mediated oocyte maturation, PI3K-Akt signaling pathway, hepatitis C, thyroid hormone signaling pathway, prolactin signaling pathway, thyroid cancer, MAPK signaling pathway, and others. Genes PIK3R1, MAPK1, SRC, RXRA, and TP53 are involved in the thyroid hormone signaling pathway and are the top targets affected by PBDEs based on the analysis of the key target in Section 5.2. PBDEs have a strong effect on the thyroid hormone signaling pathway. Meanwhile, pathways in cancer, proteoglycans in cancer, and progesterone-mediated oocyte maturation are newly discovered pathways affected by PBDEs.

The PI3K-Akt signaling pathway, thyroid hormone signaling pathway, and MAPK signaling pathway have been reported in previous studies. PBDEs inhibited the protein expression of estrogen receptor ER and GPR30 in hypothyroidism rats and further affected the expression of proteins in the PI3K/Akt and ERK1/2 signaling pathways [42]. BDE-100 has antithyroid and antiestrogenic effects and could regulate the endocrine system in a variety of ways by simultaneously interfering with multiple hormone signaling pathways [43]. PBDE mixtures including BDE-47, BDE-99, BDE-153, and other homologues activate the MAPK pathway at a concentration with no obvious cytotoxicity. The results suggest that disturbed intracellular signal transduction, including the MAPK pathway, participates in the production of adverse reactions to persistent organic pollutants [44]. At present, there are relatively few studies of PBDEs on various pathways, and further research will be needed.

### 5.4. Molecular Docking Verification

The docking results show that the hydroxylated metabolites of PBDEs could produce more hydrogen bonding interactions and hydrophobic interactions than those of PBDE prototypes, and the binding activity was stronger. In addition, PBDE sulfate metabolites generally have more hydrogen bonds and lower binding energy than hydroxylated metabolites. This indicates that PBDE sulfate metabolites have a stronger binding activity to the five thyroid function-related targets than PBDE hydroxylated metabolites, which might be an important component causing thyroid hormone endocrine disruption.

Systematic molecular docking generally believes that the binding energy between the molecule and the target has three levels: less than -4.25 kcal·mol^−1^ indicates a certain binding activity, less than -5.0 kcal·mol^−1^ indicates good binding activity, and less than -7.0 kcal·mol^−1^ indicates strong binding activity [33]. The molecular docking results show that five PBDE monomer homologues and the corresponding hydroxylated and sulfate metabolites all have a good binding affinity with the targets, which further confirms the prediction of network pharmacology. It has been reported that the toxicity of PBDE hydroxylated metabolites is stronger than that of PBDE prototypes, and some of the toxicity of PBDEs might be caused by hydroxyl metabolites [45]. The exposure of PBDE hydroxylated metabolites has a higher potential of endocrine destruction than that of prototypes [46]. Studies have confirmed that PBDE hydroxylated metabolites have a higher structural similarity with T4, resulting in a stronger regulatory effect on thyroid hormones than prototypes [47, 48]. This suggests that attention needs to be paid to hydroxylated and sulfate metabolites in the study of PBDE-thyroid hormone interference.

## 6. Conclusion

The toxicity of the PBDE mixture is different from that of its monomer, and thus, a holistic study was carried out by network pharmacology. The results show that the top five targets PIK3R1, MAPK1, SRC, RXRA, and TP53 are concentrated in the thyroid hormone signaling pathway, which preliminarily indicates that PBDEs have a strong endocrine disrupting effect on thyroid hormones. Meanwhile, pathway analysis identified 62 pathways mainly related to the regulation of the thyroid hormone signaling pathway, MAPK signaling pathway, PI3K-Akt signaling, pathways in cancer, proteoglycans in cancer, and progesterone-mediated oocyte maturation, the last three of which are newly discovered pathways affected by PBDEs. The molecular docking results show that the selected PBDE prototypes and hydroxylated and sulfate metabolites could bind to the targets, and the binding effect was enhanced in turn. The research suggests that the study of pathways affected by PBDEs can be further explored in follow-up research, and more attention should be given to PBDE metabolites.

## Data Availability

The data used to support the findings of this study are included within the article and available from the corresponding author upon request.

## Abbreviations

BP: Biological process
CC: Cellular component
MAPK1: Mitogen-activated protein kinase 1
MF: Molecular function
PBDEs: Polybrominated diphenyl ethers
PIK3R1: Phosphoinositide-3-kinase regulatory subunit 1
PPI: Protein-protein interaction
PTC: Thyroid papillary carcinoma
RXRA: Retinoid X receptor alpha
SRC: SRC proto-oncogene, nonreceptor tyrosine kinase
T3: Triiodothyronine
T4: Thyroxine
TBG: Thyroid-binding globulin
TBPA: Thyroxine-binding prealbumin
TP53: Tumor protein p53
TRs: Thyroid receptors
TTR: Transthyretin.

## Supplementary Materials

Supplementary 1 Table S1: PBDE corresponding SMILES structural formulas.

Supplementary 2 Table S2: compound target names.

Supplementary 3 Figure S1: PBDE general structure (*m* + *n* = 1‐10).

Supplementary 4 Figure S2: Venn diagram of PBDE targets and thyroid dysfunction targets.

Supplementary 5 Figure S3: protein-protein interaction network.

Supplementary 6 Figure S4: a detailed map of the thyroid hormone signaling pathway. The red part is the screened target involved in the thyroid hormone signaling pathway.

Supplementary 7 File S1: molecular docking of PBDE prototypes with key targets.

Supplementary 8 File S2: molecular docking of PBDE hydroxylated metabolites with key targets.

Supplementary 9 File S3: molecular docking of PBDE sulfate metabolites with key targets.

Supplementary 10 File S4: molecular docking of the natural ligand with key targets.

Supplementary 11 Table S3: information of targets and ligands.

Supplementary 12 Figure S5: 3D visual analysis results of molecular docking of PBDE prototypes, hydroxylated metabolites, and sulfate metabolites with key targets.

Supplementary 13 Figure S6: the 2D and 3D visual analysis results of molecular docking of the natural ligand with key targets.
