# Supplementary material for: Combining Network Pharmacology with Molecular Docking for Mechanistic Research on Thyroid Dysfunction Caused by Polybrominated Diphenyl Ethers and Their Metabolites
Source: Biomed Res Int. 2021 Nov 17;2021:2961747. doi: 10.1155/2021/2961747 (PMC8613503; doi:10.1155/2021/2961747)
Supplement: Supplementary 1 — Table S1: PBDE corresponding SMILES structural formulas. [file 2961747.f1.docx]

**Table S1. PBDEs corresponding SMILES structural formulas**

| **compound names** | **SMILES structural formulas** |
| --- | --- |
| BDE-17 | C1=CC=C(C(=C1)OC2=C(C=C(C=C2)Br)Br)Br |
| BDE-28 | C1=CC(=CC=C1OC2=C(C=C(C=C2)Br)Br)Br |
| BDE-47 | C1=CC(=C(C=C1Br)Br)OC2=C(C=C(C=C2)Br)Br |
| BDE-49 | C1=CC(=C(C=C1Br)Br)OC2=C(C=CC(=C2)Br)Br |
| BDE-66 | C1=CC(=C(C=C1OC2=C(C=C(C=C2)Br)Br)Br)Br |
| BDE-85 | C1=CC(=C(C=C1Br)Br)OC2=C(C(=C(C=C2)Br)Br)Br |
| BDE-99 | C1=CC(=C(C=C1Br)Br)OC2=CC(=C(C=C2Br)Br)Br |
| BDE-100 | C1=CC(=C(C=C1Br)Br)OC2=C(C=C(C=C2Br)Br)Br |
| BDE-119 | C1=CC(=C(C=C1OC2=C(C=C(C=C2Br)Br)Br)Br)Br |
| BDE-153 | C1=C(C(=CC(=C1Br)Br)Br)OC2=CC(=C(C=C2Br)Br)Br |
| BDE-154 | C1=C(C=C(C(=C1Br)OC2=CC(=C(C=C2Br)Br)Br)Br)Br |
| BDE-183 | C1=C(C(=CC(=C1Br)Br)Br)OC2=C(C(=C(C=C2Br)Br)Br)Br |
| BDE-197 | C1=C(C(=C(C(=C1Br)Br)Br)OC2=C(C(=C(C=C2Br)Br)Br)Br)Br |
| BDE-206 | C1=C(C(=C(C(=C1Br)Br)Br)Br)OC2=C(C(=C(C(=C2Br)Br)Br)Br)Br |
| BDE-207 | C1=C(C(=C(C(=C1Br)Br)Br)OC2=C(C(=C(C(=C2Br)Br)Br)Br)Br)Br |
| BDE-209 | C1(=C(C(=C(C(=C1Br)Br)Br)Br)Br)OC2=C(C(=C(C(=C2Br)Br)Br)Br)Br |
