# Supplementary material for: Combining Network Pharmacology with Molecular Docking for Mechanistic Research on Thyroid Dysfunction Caused by Polybrominated Diphenyl Ethers and Their Metabolites
Source: Biomed Res Int. 2021 Nov 17;2021:2961747. doi: 10.1155/2021/2961747 (PMC8613503; doi:10.1155/2021/2961747)
Supplement: Supplementary 2 — Table S2: compound target names. [file 2961747.f2.docx]

**Table S2. Compound targets name**

| ABL1 | AKR1C3 | CBS | EIF4E | FECH |
| --- | --- | --- | --- | --- |
| ABO | AKT2 | CCNA2 | ELANE | FGFR1 |
| ADAM17 | ALB | CDK2 | EPHB4 | FGFR2 |
| ADH1C | ANXA5 | CES1 | ERBB4 | FKBP1A |
| ADH5 | APOA2 | CHEK1 | ESR1 | FNTA |
| ADK | AR | CRABP2 | ESR2 | GART |
| AKR1B1 | AURKA | CTNNA1 | ESRRA | GC |
| AKR1C1 | BACE1 | CTSB | ESRRG | GLO1 |
| AKR1C2 | BCL2L1 | CTSK | F10 | GM2A |
| HMGCR | BHMT | CTSS | F11 | GRB2 |
| HMOX1 | BIRC7 | CYP2C8 | F2 | GSK3B |
| HNF4G | BLVRB | CYP2C9 | F7 | GSTA1 |
| HNMT | CA2 | DCK | FABP3 | GSTP1 |
| HSD11B1 | CASP1 | DHODH | FABP5 | GSTT2B |
| HSD17B1 | CASP3 | DPEP1 | FABP6 | HCK |
| HSP90AB1 | CASP7 | DPP4 | FABP7 | HDAC8 |
| IGF1R | CBR1 | EGFR | LCN2 | SDS |
| IL2 | NR1I2 | PLA2G10 | LTA4H | SEC14L2 |
| INSR | NR1I3 | PLA2G2A | MAOA | SERPINA1 |
| ITGAL | NR3C2 | PNMT | MAOB | SHBG |
| ITK | PADI4 | PPARA | MAP2K1 | SHMT1 |
| JAK2 | PCTP | PPARD | MAPK10 | SOD2 |
| JAK3 | PDE4B | PPARG | MAPKAPK2 | STS |
| KDR | PDE4D | PPP1CC | MDM2 | SULT1E1 |
| KIF11 | PDE5A | PRKACA | MET | SULT2A1 |
| KIT | PDK2 | PRKCQ | MIF | SULT2B1 |
| LCK | PDPK1 | PROCR | MME | SYK |
| FKBP1B | PGR | PSAP | MMP12 | TEK |
| FKBP3 | PIK3CG | PTPN1 | MMP13 | TGFB2 |
| MMP9 | PIM1 | PTPN11 | MMP2 | TGFBR1 |
| PARP1 | RFK | PYGL | MMP3 | TGM3 |
| TNNC1 | CFB | RARA | MMP8 | THRA |
| AMD1 | CSK | RARB | MTAP | THRB |
| CDK5R1 | CTSL | RARG | NQO1 | TPSB2 |
| CDK6 | AHR | RBP4 | NQO2 | TRAPPC3 |
| CFD | ALOX15 | REN | NR1H2 | TTPA |
| PLK1 | PTP4A3 | RORA | NR1H3 | TTR |
| EPHX2 | [NCEH1](http://zinc15.docking.org/genes/NCEH1) | RXRB | NR1H4 | TYMS |
| GCK | ALOX12 | S100A9 | CASP9 | VDR |
| HSPA8 | CYP2B6 | AKR1A1 | SLCO1B3 | WAS |
| IVD | PPIG | Q96JD6 | CASP8 | XIAP |
| PCK1 | CYP1A1 | FLT1 | SLCO1B1 | ZAP70 |
| PLAT | CEBPG | FLT4 | SLCO2B1 | ADAM33 |
| GFER | IL8 | SLC6A2 | CYP17A1 | CTSF |
| SLC6A3 | ATP2A1 | SLC6A4 | TDP1 | DHFR |
| SLC6A7 | MAPT | ALOX5 | P18054 | AKR1B15 |
| SLC6A14 | SLC6A9 | ALOXE3 | ALOX15B | AKR1B10 |
| SLC6A5 | O75342 |  |  |  |
