# Supplementary figures and images for: Combining Network Pharmacology with Molecular Docking for Mechanistic Research on Thyroid Dysfunction Caused by Polybrominated Diphenyl Ethers and Their Metabolites
Source: Biomed Res Int. 2021 Nov 17;2021:2961747. doi: 10.1155/2021/2961747 (PMC8613503; doi:10.1155/2021/2961747)

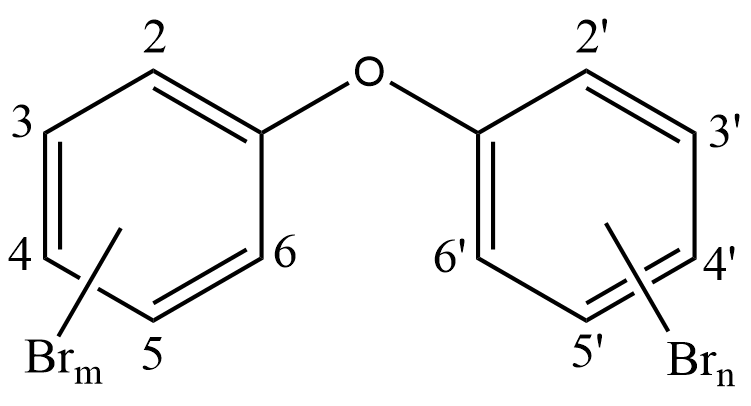


**Figure S1. PBDEs general structure (m+n=1-10)**

Supplement: Supplementary 3 — Figure S1: PBDE general structure (m + n = 1‐10). [file 2961747.f3.docx]

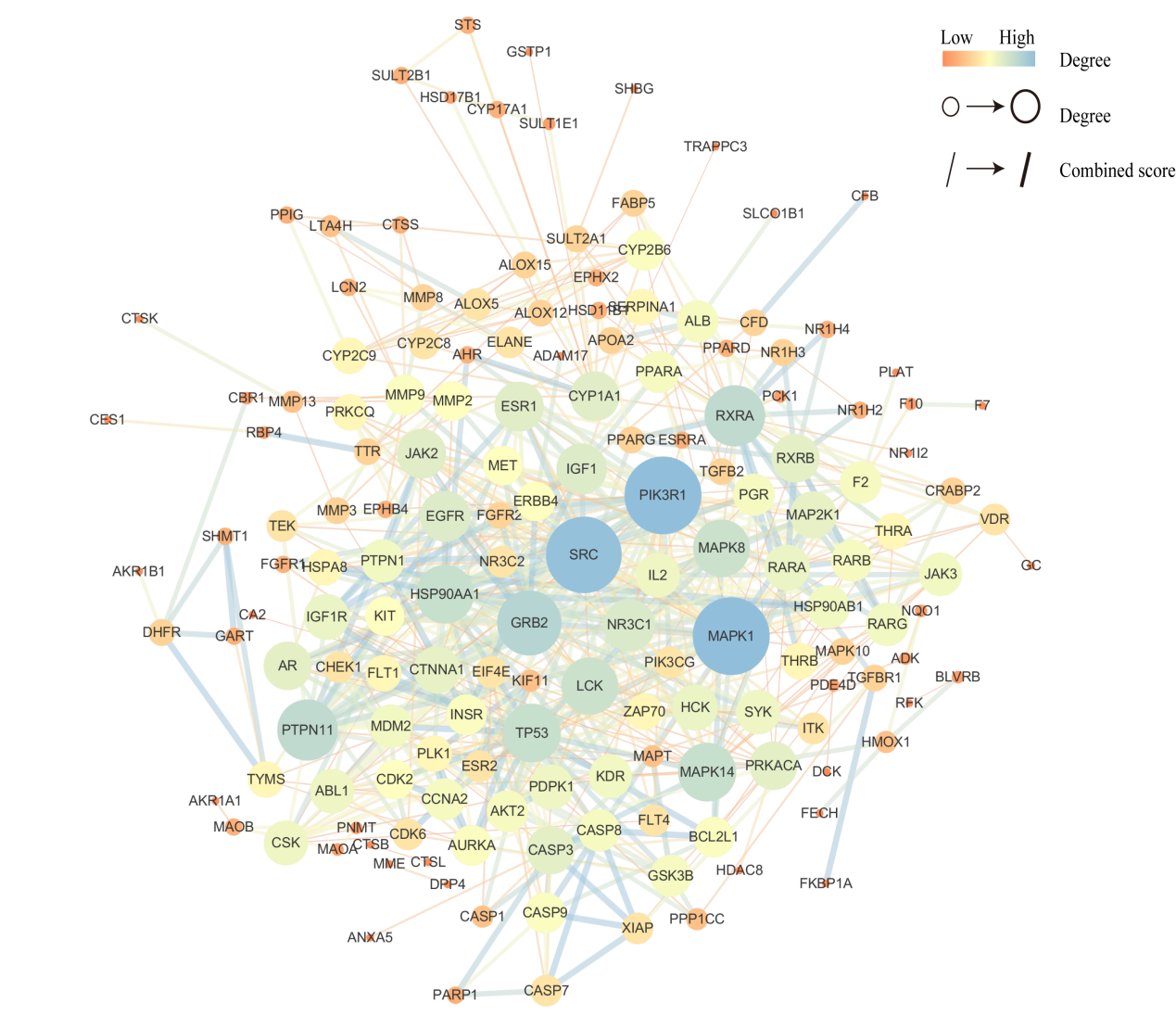


**Figure S3. Protein-protein interaction network**

Supplement: Supplementary 5 — Figure S3: protein-protein interaction network. [file 2961747.f5.docx]
