# Supplementary material for: Combining Network Pharmacology with Molecular Docking for Mechanistic Research on Thyroid Dysfunction Caused by Polybrominated Diphenyl Ethers and Their Metabolites
Source: Biomed Res Int. 2021 Nov 17;2021:2961747. doi: 10.1155/2021/2961747 (PMC8613503; doi:10.1155/2021/2961747)
Supplement: Supplementary 4 — Figure S2: Venn diagram of PBDE targets and thyroid dysfunction targets. [file 2961747.f4.docx]

**Figure S2. Venn diagram of PBDEs targets and thyroid dysfunction targets**
