# Supplementary material for: Combining Network Pharmacology with Molecular Docking for Mechanistic Research on Thyroid Dysfunction Caused by Polybrominated Diphenyl Ethers and Their Metabolites
Source: Biomed Res Int. 2021 Nov 17;2021:2961747. doi: 10.1155/2021/2961747 (PMC8613503; doi:10.1155/2021/2961747)
Supplement: Supplementary 6 — Figure S4: a detailed map of the thyroid hormone signaling pathway. The red part is the screened target involved in the thyroid hormone signaling pathway. [file 2961747.f6.docx]

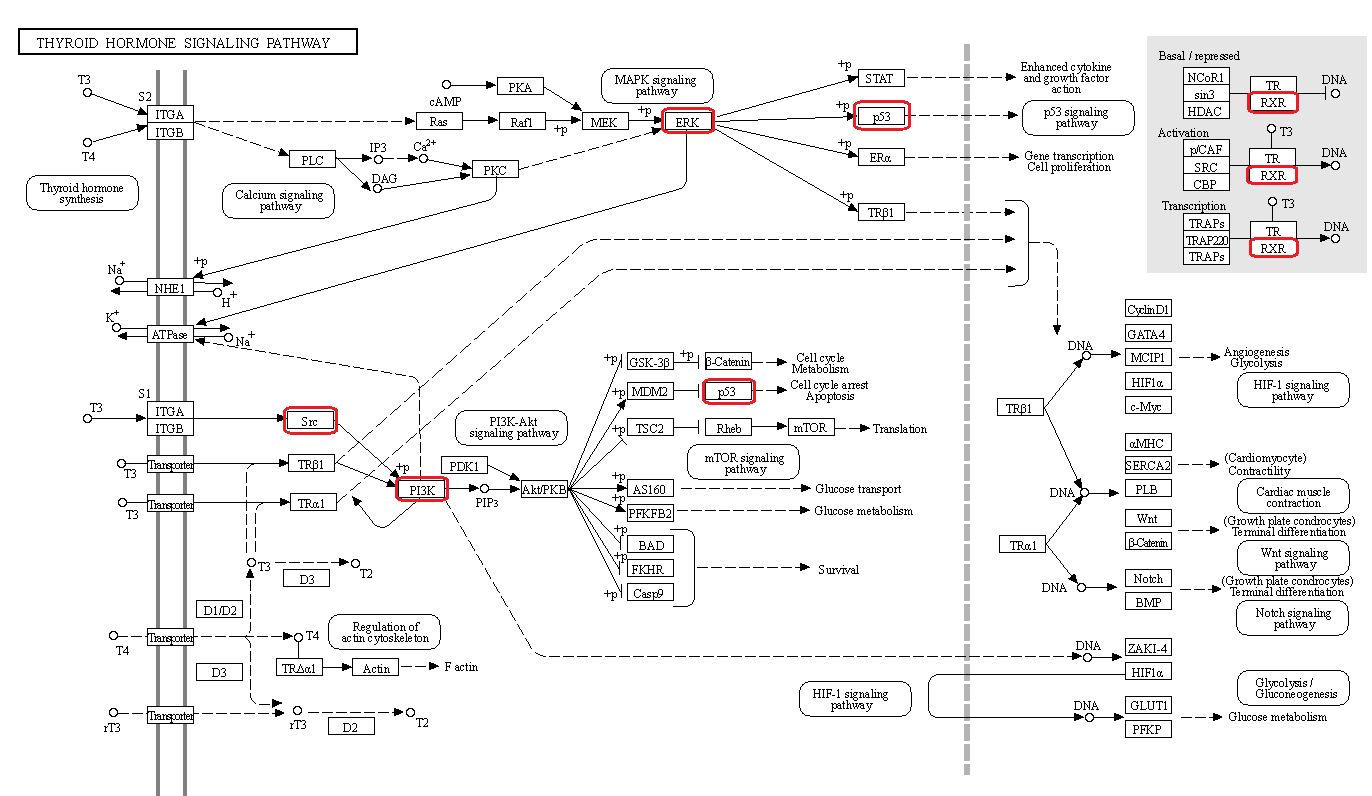


**Figure S4. A detailed map of the thyroid hormone signaling pathway. The red part is the screened target involved in the thyroid hormone signaling pathway**
