# Supplementary material for: Combining Network Pharmacology with Molecular Docking for Mechanistic Research on Thyroid Dysfunction Caused by Polybrominated Diphenyl Ethers and Their Metabolites
Source: Biomed Res Int. 2021 Nov 17;2021:2961747. doi: 10.1155/2021/2961747 (PMC8613503; doi:10.1155/2021/2961747)
Supplement: Supplementary 7 — File S1: molecular docking of PBDE prototypes with key targets. [file 2961747.f7.docx]

**File S1. Molecular docking of PBDEs prototypes with key targets**

The docking results of BDE-47 and PIK3R1 are shown (Figure S5 A2). BDE-47 formed hydrophobic interactions with the hydrophobic cavity of the amino acid residues Arg30, Ala108, Tyr104, Ala103, Pro5, Pro105 and Lys29 near the active site. Natural ligand PTR formed three hydrogen bond interactions with the active site of PIK3R1, a hydrogen bond interaction with the NH on the main chain of amino acid residues His85 and His88, a hydrogen bond interaction with the NH on the main chain of amino acid residue Ser77, and hydrophobic interactions with the hydrophobic cavity of four amino acid residues, Leu75, Tyr76, Glu81, and Leu84, near the active site. The docking binding energy between BDE-47 and PIK3R1 was -5.7 kcal·mol^-1^, which was lower than that of natural ligand PTR - 5.3 kcal·mol^-1^ and common standard - 5.0 kcal·mol^-1^, indicating good binding activity between BDE-47 and PIK3R1.

The docking results of BDE-99 and TP53 are shown (Figure S5 B2). BDE-99 formed a hydrogen bond interaction with the NH on the main chain of the amino acid residue Phe113. It also had hydrophobic interactions with the hydrophobic cavity of three amino acid residues, Leu111, Trp146 and Gly112, near the active site. The B loop of BDE-99 formed a π-π interaction with the amino acid residue Trp146. Meanwhile, the natural ligand EY2 formed a hydrogen bond interaction with the C=O on the main chain of the amino acid residue Phe113. It also had hydrophobic interactions with the hydrophobic cavity formed by the 8 amino acid residues Ser269, Tyr126, Asn131, Asp268, Pro128, Leu111, Gly112 and His115 near the active site. Amino acid residues Leu111 and Gly112 were the common amino acid residues for the docking of BDE-99 and natural ligand EY2 with the TP53. The docking binding energy between BDE-99 and TP53 was -5.5 kcal·mol^-1^, which was higher than that of natural ligands EY2 and TP53 (-5.9 kcal·mol^-1^) and common standard (-5.0 kcal·mol^-1^).

The docking results of BDE-153 and SRC are shown (Figure S5 D2). BDE-153 had hydrophobic interactions with the hydrophobic cavity of five amino acid residues, Cys280, Ala406, Leu276, Val284 and Leu396 near the active site. The natural ligand HVY formed a hydrogen bond interaction with the NH on the main chain of amino acid residue Glu356 and formed hydrophobic interactions with the hydrophobic cavity of fifteen amino acid residues, Ala296, Leu396, Leu276, Gly277, Gln278, Val284, Met344, Gly347, Lys346, Phe352, Ala393, Asp351, Ser348, Gly355 and Thr357, near the active site. Amino acid residues Leu276, Val284, and Leu396 were the common amino acid residues for the docking of BDE-153 and natural ligand HVY with SRC. The docking binding energy of BDE-153 and SRC was -7.8 kcal·mol^-1^, which was higher than the docking binding energy of natural ligands HVY and SRC of -9.1 kcal·mol^-1^ and the common standard of -5.0 kcal·mol^-1^.

The docking results of BDE-153 and MAPK1 are shown (Figure S5 E2). BDE-153 had hydrophobic interactions with two amino acid residues, Lys54 and Gln105, near the active site. The natural ligand FRZ formed hydrophobic interactions with the hydrophobic cavity of nine amino acid residues Ala52, Gln105, Ile103, Lys54, Asp167, Tyr36, Asp111, Leu156, and Val39 near the active site. Amino acid residue Lys54 was the common amino acid residue for the docking of BDE-153 and natural ligand FRZ with the MAPK1. The docking binding energy of BDE-153 with MAPK1 was -6.6 kcal·mol^-1^, which was higher than the natural ligand FRZ with MAPK1 of -8.7 kcal·mol^-1^ and the common standard of -5.0 kcal·mol^-1^.
