# Supplementary material for: Combining Network Pharmacology with Molecular Docking for Mechanistic Research on Thyroid Dysfunction Caused by Polybrominated Diphenyl Ethers and Their Metabolites
Source: Biomed Res Int. 2021 Nov 17;2021:2961747. doi: 10.1155/2021/2961747 (PMC8613503; doi:10.1155/2021/2961747)
Supplement: Supplementary 8 — File S2: molecular docking of PBDE hydroxylated metabolites with key targets. [file 2961747.f8.docx]

**File S2. Molecular docking of PBDEs hydroxylated metabolites with key targets**

The docking results of 6-OH-BDE47 and SRC are shown (Figure S5 F2). 6-OH-BDE47 formed a hydrogen bond interaction with the OH on the main chain of the amino acid residue Thr341 and hydrophobic interactions with the hydrophobic cavity of the amino acid residues Ala296, Val284, Leu396, Asp407, Ala406, Glu313, Val326, Met317 and Phe408 near the active site. On the other hand, the natural ligand HVY formed a hydrogen bond interaction with the NH on the main chain of amino acid residue Glu356 and hydrophobic interactions with the hydrophobic cavity of the surrounding 15 amino acid residues Ala296, Leu396, Leu276, Gly277, Gln278, Val284, Met344, Gly347, Lys346, Phe352, Ala393, Asp351, Ser348, Gly355 and Thr357 near the active site. Among them, amino acid residues Ala296, Val284 and Leu396 were the common amino acid residues for 6-OH-BDE47 and natural ligand HVY. The docking binding energy of 6-OH-BDE47 and SRC was -7.3 kcal·mol^-1^ less than -5.0 kcal·mol^-1^ but higher than HVY -9.1 kcal·mol^-1^.

The docking results of 3-OH-BDE47 and MAPK1 are shown (Figure S5 H2). 3-OH-BDE47 formed two hydrogen bond interactions with C=O and NH on the main chain of the amino acid residue Met108. It also had hydrophobic interactions with the hydrophobic cavity of seven amino acid residues, Ala52, Ile31, Cys166, Leu107, Leu156, Val39 and Lys54, near the active site. The natural ligand FRZ formed hydrophobic interactions with the hydrophobic cavity of nine amino acid residues, Ala52, Gln105, Ile103, Lys54, Asp167, Tyr36, Asp111, Leu156, and Val39, near the active site. Among them, amino acid residues Ala52, Leu156, Val39 and Lys54 were the common amino acid residues for the docking of 3-OH-BDE47 and natural ligand FRZ with MAPK1. The docking binding energy of 3-OH-BDE47 and MAPK1 was -6.7 kcal·mol^-1^ less than -5.0 kcal·mol^-1^ but higher than that of the natural ligands FRZ and MAPK1 (-8.7 kcal·mol^-1^).

The docking results of 5-OH-BDE99 and TP53 are shown (Figure S5 I2). 5-OH-BDE99 formed a hydrogen bond interaction with the C=O on the main chain of amino acid residue Leu145 and had hydrophobic interactions with the hydrophobic cavity of five amino acid residues, Cys229, Val147, Pro223, Trp146 and Ser227, near the active site. The natural ligand EY2 formed a hydrogen bond interaction with the C=O on the main chain of the amino acid residue Phe113 and hydrophobic interactions with the hydrophobic cavity of eight amino acid residues, Ser269, Tyr126, Asn131, Asp268, Pro128, Leu111, Gly112 and His115, near the active site. The docking binding energy of 5-OH-BDE99 and TP53 was -5.9 kcal·mol^-1^ less than -5.0 kcal·mol^-1^, which was the same as that of the natural ligand EY2.

The docking results of 5’-OH-BDE99 and RXRA are shown (Figure S5 J2). 5'-OH-BDE99 formed a hydrogen bond interaction with the C=O on the main chain of amino acid residue Ile268 and had hydrophobic interactions with the hydrophobic cavity of six amino acid residues, Gln275, Ala271, Phe313, Ala272, Leu309 and Cys432, near the active site. The natural ligand BM6 had hydrophobic interactions with the hydrophobic cavity of eight amino acid residues, Glu453, Thr449, Phe450, Leu301, Val298, Leu294, Val280 and Phe277. The docking binding energy of 5'-OH-BDE99 and RXRA was -8.2 kcal·mol^-1^ less than -5.0 kcal·mol^-1^ but higher than that of the natural ligands BM6 and RXRA (-8.9 kcal·mol^-1^).
