# Supplementary material for: Combining Network Pharmacology with Molecular Docking for Mechanistic Research on Thyroid Dysfunction Caused by Polybrominated Diphenyl Ethers and Their Metabolites
Source: Biomed Res Int. 2021 Nov 17;2021:2961747. doi: 10.1155/2021/2961747 (PMC8613503; doi:10.1155/2021/2961747)
Supplement: Supplementary 9 — File S3: molecular docking of PBDE sulfate metabolites with key targets. [file 2961747.f9.docx]

**File S3. Molecular docking of PBDEs sulphate metabolites with key targets**

The docking results of 6-BDE47 sulphate and MAPK1 are shown (Figure S5 K2). 6-BDE47 sulphate formed a hydrogen bonding interaction with the NH on the main chain of amino acid residue Glu334 and two hydrogen bonding interactions with the two NH2 around C6 on the main chain of amino acid residue Arg70. It also had hydrophobic interactions with the hydrophobic cavity of the amino acid residues Phe331, Val173, Arg172 and Leu335. The natural ligand FRZ formed hydrophobic interactions with the hydrophobic cavity of the surrounding amino acid residues Ala52, Gln105, Ile103, Lys54, Asp167, Tyr36, Asp111, Leu156, and Val39 near the active site. The docking binding energy of 6-BDE47 sulphate and MAPK1 was - 7.7 kcal·mol^-1^ less than - 5.0 kcal·mol^-1^ but higher than the natural ligand FRZ (-8.7 kcal·mol^-1^).

The docking results of 3-BDE47 sulphate and RXRA are shown (Figure S5 M2). 3-BDE47 sulphate had hydrophobic interactions with the hydrophobic cavity of ten amino acid residues, His435, Ile345, Val342, Phe439, Cys432, Ala272, Leu309, Gln275, Ile268 and Phe313, near the active site. The natural ligand BM6 had hydrophobic interactions with the hydrophobic cavity of eight amino acid residues, Glu453, Thr449, Phe450, Leu301, Val298, Leu294, Val280 and Phe277. The docking binding energy of 3-BDE47 sulphate and RXRA was -8.7 kcal· mol^-1^ less than -5.0 kcal·mol^-1^ but slightly higher than that of the natural ligand BM6 (-8.9 kcal·mol^-1^).

The docking results of 5-BDE99 sulphate and SRC are shown (Figure S5 N2). 5-BDE99 sulphate formed a hydrogen bonding interaction with the OH on the main chain of amino acid residue Thr341 and formed hydrophobic interactions with the hydrophobic cavity of eight amino acid residues, Ala296, Val284, Lys298, Ile297, Leu276, Leu396, Ala406 and Cys280, near the active site. The natural ligand HVY had a hydrogen bond interaction with the NH on the main chain of amino acid residue Glu356, and also had hydrophobic interactions with the hydrophobic cavity of fifteen amino acid residues, Ala296, Leu396, Leu276, Gly277, Gln278, Val284, Met344, Gly347, Lys346, Phe352, Ala393, Asp351, Ser348, Gly355 and Thr357, near the active site. The amino acid residues Ala296, Val284, Leu276 and Leu396 were the common amino acid residues for 5-BDE99 sulphate and natural ligand HVY. The docking binding energy of 5-BDE99 sulphate and SRC was -8.2 kcal·mol^-1^ , which was less than -5.0 kcal·mol^-1^ but higher than that of the natural ligand HVY (-9.1 kcal·mol^-1^).

The docking results of 5’-BDE99 sulphate and TP53 are shown (Figure S5 O2). 5'-BDE99 sulphate formed a hydrogen bonding interaction with the OH on the main chain of amino acid residue Tyr126 and a hydrogen bonding interaction with the NH on the main chain of amino acid residue Phe113. It also had hydrophobic interactions with the amino acid residues Leu111, Trp146 and Gly112. The natural ligand EY2 formed a hydrogen bond interaction with the C=O on the main chain of the amino acid residue Phe113 and hydrophobic interactions with the hydrophobic cavity formed by the 8 amino acid residues Ser269, Tyr126, Asn131, Asp268, Pro128, Leu111, Gly112 and His115 near the active site. The amino acid residues Leu111 and Gly112 were the common amino acid residues for BDE-99 and natural ligand EY2 with TP53. The docking binding energy of 5'-BDE99 sulphate and TP53 was -6.4 kcal·mol^-1^, less than -5.0 kcal·mol^-1^, which was also lower than that of the natural ligand EY2 (-5.9 kcal·mol^-1^).
