# Supplementary material for: Combining Network Pharmacology with Molecular Docking for Mechanistic Research on Thyroid Dysfunction Caused by Polybrominated Diphenyl Ethers and Their Metabolites
Source: Biomed Res Int. 2021 Nov 17;2021:2961747. doi: 10.1155/2021/2961747 (PMC8613503; doi:10.1155/2021/2961747)
Supplement: Supplementary 10 — File S4: molecular docking of the natural ligand with key targets. [file 2961747.f10.docx]

**File S4. Molecular docking of the natural ligand with key targets**

The natural ligand PTR formed three hydrogen bond interactions with the active site of PIK3R1, a hydrogen bond interaction with the NH on the main chain of amino acid residues His85 and His88, a hydrogen bond interaction with the NH on the main chain of amino acid residue Ser77, and hydrophobic interactions with the hydrophobic cavity of four amino acid residues, Leu75, Tyr76, Glu81, and Leu84, near the active site(Figure S6 A3 and B3).

The natural ligand FRZ formed hydrophobic interactions with the hydrophobic cavity of nine amino acid residues, Ala52, Gln105, Ile103, Lys54, Asp167, Tyr36, Asp111, Leu156, and Val39 near the active site (Figure S6 C3 and D3).

The natural ligand HVY formed a hydrogen bond interaction with the NH on the main chain of amino acid residue Glu356 and formed hydrophobic interactions with the hydrophobic cavity of fifteen amino acid residues, Ala296, Leu396, Leu276, Gly277, Gln278, Val284, Met344, Gly347, Lys346, Phe352, Ala393, Asp351, Ser348, Gly355 and Thr357, near the active site respectively(Figure S6 E3 and F3).

The natural ligand BM6 had hydrophobic interactions with the hydrophobic cavity of eight amino acid residues, Glu453, Thr449, Phe450, Leu301, Val298, Leu294, Val280 and Phe277 (Figure S6 G3 and H3).

The natural ligand EY2 formed a hydrogen bond interaction with the C=O on the main chain of the amino acid residue Phe113. It also had hydrophobic interactions with the hydrophobic cavity formed by the 8 amino acid residues Ser269, Tyr126, Asn131, Asp268, Pro128, Leu111, Gly112 and His115 near the active site(Figure S6 I3 and J3).
