# Supplementary material for: Combining Network Pharmacology with Molecular Docking for Mechanistic Research on Thyroid Dysfunction Caused by Polybrominated Diphenyl Ethers and Their Metabolites
Source: Biomed Res Int. 2021 Nov 17;2021:2961747. doi: 10.1155/2021/2961747 (PMC8613503; doi:10.1155/2021/2961747)
Supplement: Supplementary 11 — Table S3: information of targets and ligands. [file 2961747.f11.docx]

**Table S3. Information of targets and ligands**

| Uniprot-ID | Gene symbol | PDB ID | Ligand |
| --- | --- | --- | --- |
| P27986 | PIK3R1 | 1H9O | PTR |
| P28482 | MAPK1 | 1TVO | FRZ |
| P12931 | SRC | 6E6E | HVY |
| P19793 | RXRA | 1MVC | BM6 |
| P04637 | TP53 | 6GGA | EY2 |
