# Supplementary material for: Combining Network Pharmacology with Molecular Docking for Mechanistic Research on Thyroid Dysfunction Caused by Polybrominated Diphenyl Ethers and Their Metabolites
Source: Biomed Res Int. 2021 Nov 17;2021:2961747. doi: 10.1155/2021/2961747 (PMC8613503; doi:10.1155/2021/2961747)
Supplement: Supplementary 12 — Figure S5: 3D visual analysis results of molecular docking of PBDE prototypes, hydroxylated metabolites, and sulfate metabolites with key targets. [file 2961747.f12.docx]

| 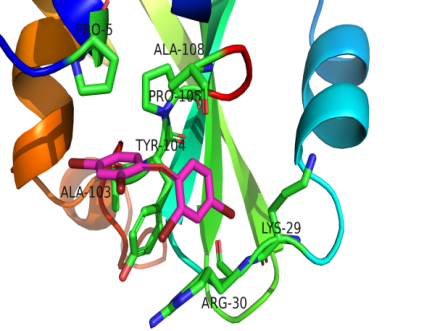  A2 | 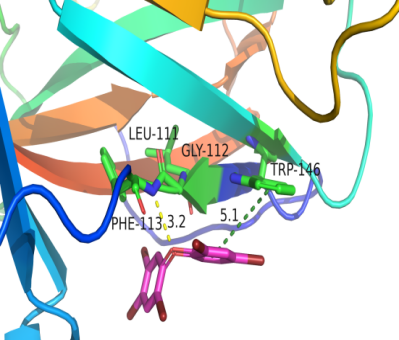  B2 | 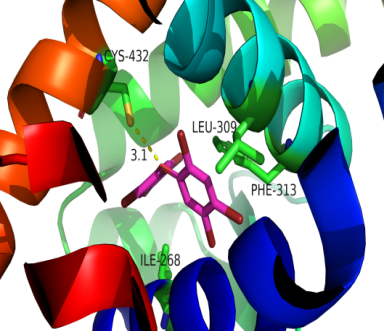  C2 |
| --- | --- | --- |
| 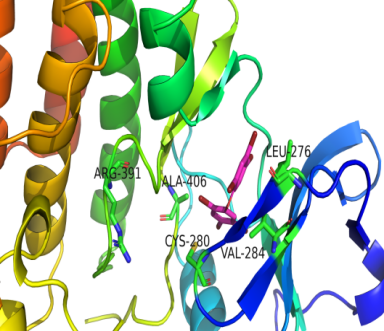  D2 | 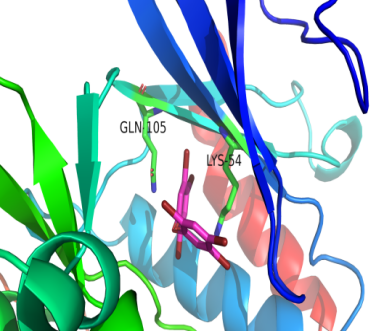  E2 | 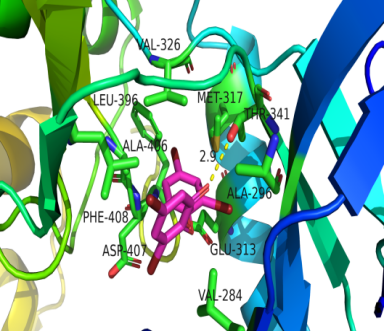  L2  I2  F2 |
| 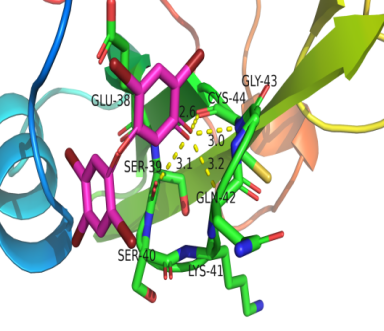  G2 | 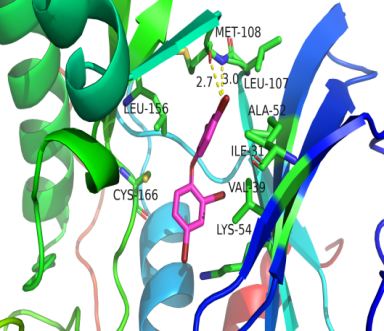  H2 | 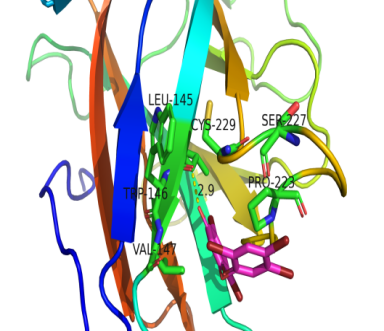 |
| 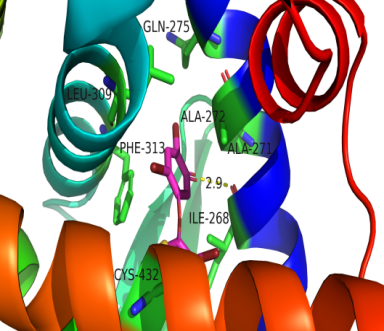  J2 | 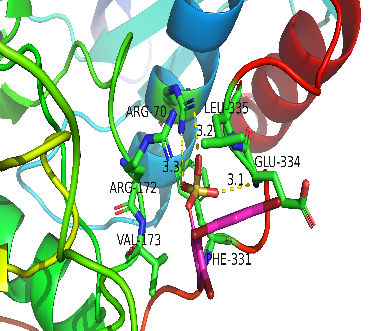  K2 | 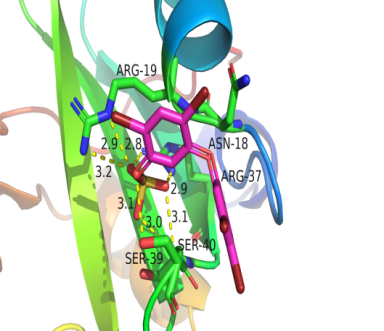 |
| 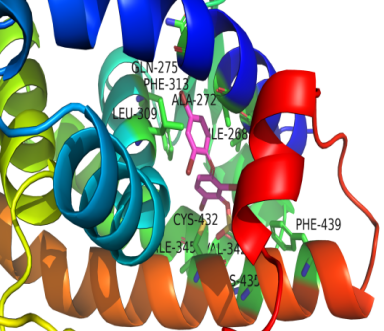  M2 | 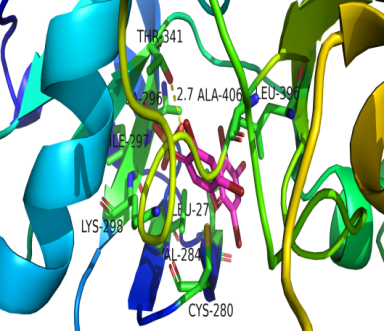  O2  N2 | 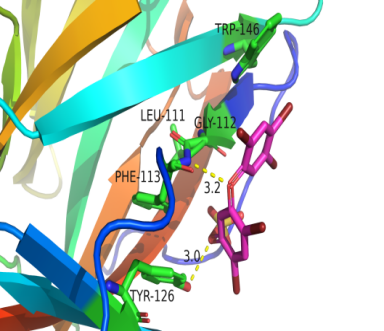 |
|  |  |  |

**Figure S5. 3D Visual analysis results of molecular docking of PBDEs prototypes, hydroxylated metabolites and sulfate metabolites with key targets(Polar interactions between the ligand (purple) and the receptor residues (green) are presented in yellow dotted lines, and the π-π interaction is presented in green dotted line.)**
