# Supplementary material for: Combining Network Pharmacology with Molecular Docking for Mechanistic Research on Thyroid Dysfunction Caused by Polybrominated Diphenyl Ethers and Their Metabolites
Source: Biomed Res Int. 2021 Nov 17;2021:2961747. doi: 10.1155/2021/2961747 (PMC8613503; doi:10.1155/2021/2961747)
Supplement: Supplementary 13 — Figure S6: the 2D and 3D visual analysis results of molecular docking of the natural ligand with key targets. [file 2961747.f13.docx]

| 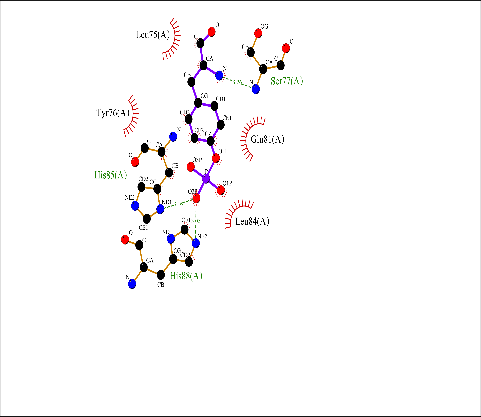  A3 | 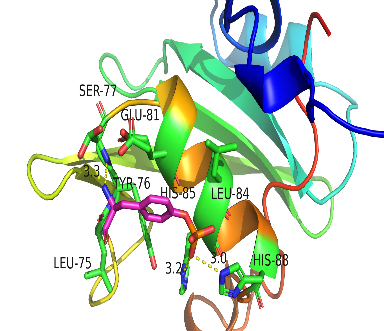  B3 |
| --- | --- |
| 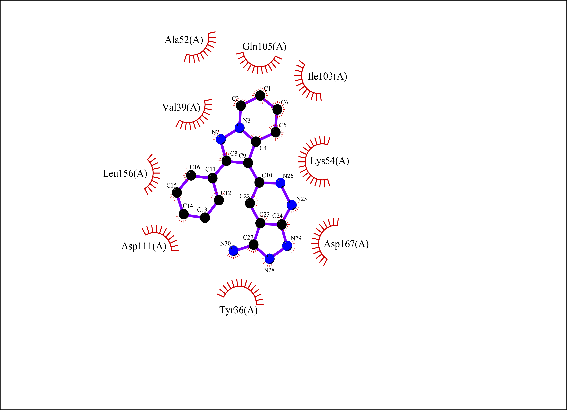  C3 | 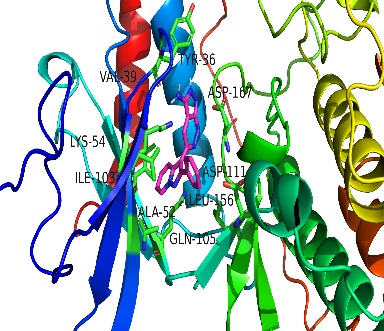  D3 |
| 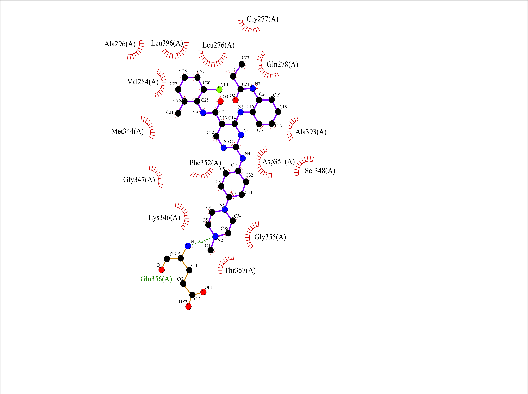  F3  E3 | 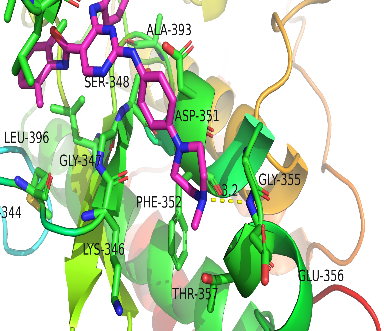 |
| 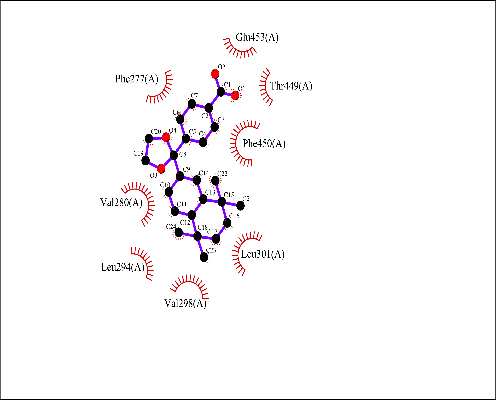  G3 | 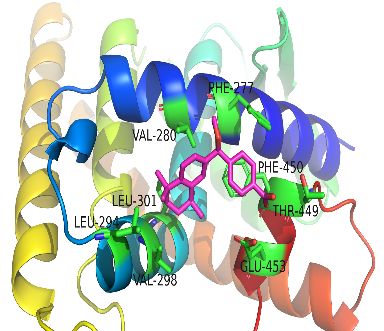  H3 |
| 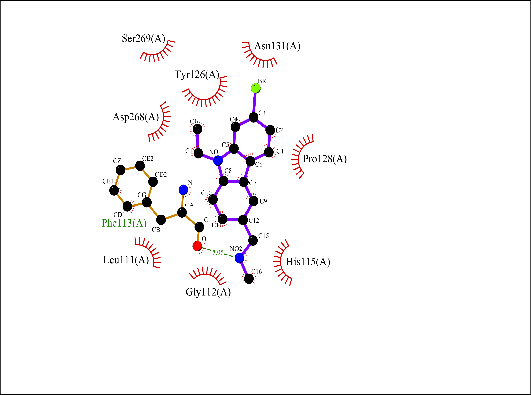  I3 | 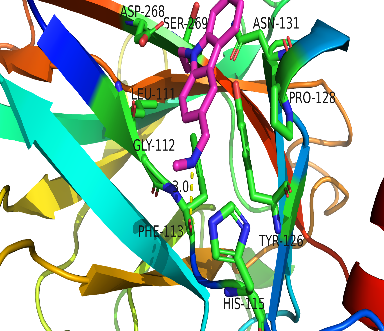  J3 |

**Figure S6. The 2D and 3D visual analysis results of molecular docking of the natural ligand with key targets (
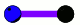
Ligand bond,
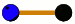
Non-ligand bond,
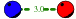
Hydrogen bond and its length,
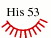
Non-ligand residues involved in hydrophobic contact(s),
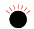
Corresponding atoms involved in hydrophobic contact(s), Polar interactions between the ligand (purple) and the receptor residues (green) are presented in yellow dotted lines)**
